# Identification of Recombinant Adeno-Associated Virus Serotypes by Matrix-Assisted Laser Desorption/Ionization Mass Spectrometry

**DOI:** 10.1021/acs.analchem.5c05430

**Published:** 2026-03-01

**Authors:** Ryoji Nakatsuka, Kenjiro Matsumoto, Yannan Liu, Kimitoshi Takeda, Yasuo Tsunaka, Tetsuo Torisu, Yuki Yamaguchi, Susumu Uchiyama

**Affiliations:** † Department of Biotechnology, Graduate School of Engineering, 13013 The University of Osaka, 2-1 Yamadaoka, Suita, Osaka 565-0871, Japan; ‡ Technology Research Laboratory, Shimadzu Corporation, 1 Nishinokyo-Kuwabaracho, Nakagyo-ku, Kyoto 604-8511, Japan; § The University of Osaka Shimadzu Analytical Innovation Research Laboratories, The University of Osaka, 2-1 Yamadaoka, Suita, Osaka 565-0871, Japan; ∥ 718133 U-Medico Inc., 2-1 Yamadaoka, Suita, Osaka 565-0871, Japan

## Abstract

Recombinant adeno-associated viruses (rAAVs) are widely used as vectors for gene therapy because they can target specific tissues, exhibit low immunogenicity, and have the capacity for long-term transgene expression. The rAAV serotype determines its tropism, therapeutic efficacy, and suitability for specific clinical applications. Moreover, new mutant serotypes have emerged through capsid engineering approaches. Accurate serotype identification is therefore essential for optimizing vector design and ensuring successful therapeutic outcomes. Conventional methods for identifying rAAV serotypes, such as the enzyme-linked immunosorbent assay, often involve complex procedures and rely on specialized reagents, including antibodies. Meanwhile, liquid chromatography-tandem mass spectrometry-based peptide mapping faces challenges in terms of quality control. These limitations highlight the need for more efficient and practical approaches to streamline the identification of rAAV serotypes. This report proposes a methodology for rAAV serotype identification using matrix-assisted laser desorption/ionization mass spectrometry (MALDI–MS) with new scoring algorithm. The developed method can distinguish multiple rAAV serotypes, including mutant serotypes, by analyzing the specific peptide mass fingerprints of each serotype. This approach eliminates the need for antibodies or extensive sample preparation, thereby offering a reagent-free and cost-effective alternative to conventional methods. Moreover, MALDI–MS requires small sample quantities, making it suitable for high-throughput screening, which can facilitate the development of tailored gene therapy vectors. This study highlights the importance of integrating advanced analytical technologies into gene therapy to enhance precision, reduce costs, and ultimately improve patient outcomes.

## Graphical abstract

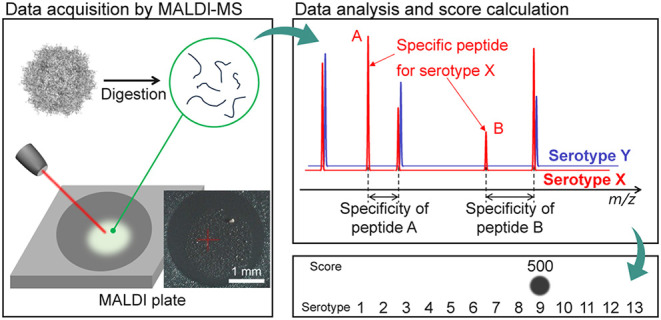

With the rapid evolution of gene therapy over the past two decades, recombinant adeno-associated virus (rAAV) has emerged as one of the most versatile and clinically translatable gene-delivery platforms.^1,2^ Multiple rAAV-based therapeutics have gained regulatory approval for inherited retinal disorders and spinal muscular atrophy, and more than 100 additional candidates are under clinical investigation for a wide range of monogenic or multifactorial diseases.^2,3^ Notably, rAAV have valuable intrinsic properties, including a broad yet tunable tissue tropism,^4–8^ fewer innate and adaptive immune responses than other viral vectors,^9^ and prolonged episomal persistence of the transgene;^10^ moreover, they can be produced at an industrial scale with acceptable purity and potency.^11,12^ The existence of numerous naturally occurring capsid serotypes and the possibility of engineering synthetic capsids allow researchers to match vector serotypes to specific therapeutic needs, thereby maximizing efficacy while minimizing off-target exposure.

In both preclinical optimization and clinical manufacturing stages, unequivocal identification of the capsid serotype is crucial to ensure quality control.^13,14^ In practical drug-development and clinical settings, identification of capsid serotype is performed as confirmatory tests to verify that the serotype produced matches that intended by the plasmid design. Traditionally, serotype confirmation relies on the enzyme-linked immunosorbent assay (ELISA), which employs monoclonal or polyclonal antibodies raised against epitopes on the viral capsid.^13,15^ Although ELISA is well established, sensitive, and compatible with most bioprocessing laboratories, this method has certain limitations. For example, antibody lots may exhibit batch-to-batch variability,^16,17^ loss of binding activity following long-term storage.^17,18^ Unforeseen cross-reactivity^19–21^ can occur among wild-type of different serotypes, and between wild-type and engineered capsids that are subtly mutated from the parent serotype. In a previous study by Mietzsch et al.,^21^ a striking result was reported: the AAV1 antibodies ADK1a cross-reacted with AAV6 and 12, the AAV2 antibody A20 cross-reacted with AAV3, and the AAV8 antibody ADK8 cross-reacted with AAV1, 3, 7, and Rh10. These findings highlight the vulnerability of identity testing by ELISA. Given such cross-reactivity, it is unlikely that ELISA assays can adequately address mutant serotypes, which are expected to become increasingly important in current and future pharmaceutical development. Additionally, ELISA-based identity testing generally necessitates the generation and qualification of variant-specific antibodies whenever new capsids are engineered. Additionally, increasing regulatory pressure, e.g., from Food and Drug Administration (FDA) encourages the reduction of animal-derived reagents in testing,^22^ thus highlighting the need for alternative serotype identification methods that are fully synthetic, reproducible, and scalable.

As an alternative approach, peptide mapping with liquid chromatography (LC) tandem mass spectrometry (MS/MS) can be used to distinguish the rAAV serotypes.^23^ This method combines proteolytic digestion with high-resolution MS/MS sequencing to afford >95% theoretical sequence coverage of VP1–3, and it can identify single amino acid substitutions introduced during directed evolution or site-directed mutagenesis.^24–28^ This depth of coverage, together with automated database searching, provides an objective molecular signature independent of antibody availability or epitope integrity. Despite these analytical advantages, LC–MS/MS is not suitable for in-process control. Nano- or microflow LC injections^24,26^ rely on fragile capillary columns and require meticulous gradient reproducibility to maintain retention-time libraries. Obtaining an annotated sequence from a thawed sample involves multiple coordinated steps and stringent controls in current GMP settings. Moreover, substantial material (typically, 10^12^–10^13^ capsids are initially required) must be injected to generate spectra with sufficient signal-to-noise ratios.^27^ Such quantities may be prohibitive during early upstream optimization or parallel screening of multiserotype panels. Finally, the costs of high-end Orbitrap or Q-TOF instruments and the need for dedicated bioinformatics pipelines, limit LC–MS/MS accessibility in many quality-control settings.

Although ELISA and LC–MS/MS are used for rAAV serotype identification, both methods have intrinsic drawbacks, all of which constrain GMP operations. Matrix-assisted laser desorption/ionization mass spectrometry (MALDI–MS) is an appealing alternative because it eliminates the need for unstable antibodies that can trigger cross-reactivity and the liquid-chromatography in LC–MS/MS workflows.^29^ Recent advances in clinical microbiology have firmly established MALDI–TOF MS as a rapid and reliable platform for microorganism identification, supported by extensive studies integrating spectral libraries with computational algorithms. Building on these developments, MALDI is increasingly recognized as a versatile tool beyond microbial diagnostics.^30,31^ The present study evaluates whether MALDI peptide-mass fingerprinting (PMF) can deliver unequivocal and robust serotyping of rAAV. In PMF, peptides generated by an enzyme are identified by their masses to yield a characteristic spectral pattern that can be matched to a reference library without the need for tandem MS. The workflow we investigate integrates a capsid reference library, enzyme selection guided by in silico digestion to enhance interserotype spectral discriminability, and automated matching of experimental PMF to the library. We assess serotype discrimination across wild-type and engineered rAAV capsids and evaluate the robustness and reproducibility of the MALDI–MS-based workflow in a GMP-relevant context.

## Experimental Section

### rAAV Preparation

Suspension-adapted HEK293F cells (Viral Production Cells 2.0, Thermo Fisher Scientific, Waltham, MA, USA) were maintained in BalanCD HEK293 medium (FUJIFILM Irvine Scientific, Santa Ana, CA, USA) supplemented with 6 mM l-glutamine (FUJIFILM, Tokyo, Japan). Cells were seeded at 1.0 × 10^6^ cells/mL, incubated at 37 °C, under 8% CO_2_, at 125 rpm, and grown to a density of 2.0 × 10^6^ cells/mL for transfection. For each serotype, cells were cotransfected with a transgene plasmid, an adenoviral helper plasmid, and a pAAV-Rep and Cap plasmid encoding the appropriate capsid genes (AAV2, AAV5, AAV6, AAV8, AAV9, AAV-PHP.eB, and AAV.GTX). Plasmids were mixed in a 1:1:1 mass ratio (total DNA, 1 μg/10^6^ cells) and complexed with FectoVIR-AAV (Polyplus, Illkirch, France). Cultures were returned to the incubator and shaken for 72 h post-transfection. After transfection, cells were lysed to release rAAV particles, which were then purified by affinity chromatography and cesium chloride density gradient ultracentrifugation in an Optima XE-90 (Beckman Coulter, Inc., Brea, CA, USA) or small-scale affinity purification as referred to our paper.^32^

### Enzymatic Digestion and Desalting

First, 24 μL of rAAV samples were placed in an incubator set at 90 °C and incubated for 10 min to induce thermal denaturation. Then, 4.1 μL of Milli-Q water and 2.5 μL of 1 M ammonium bicarbonate at pH 7.6–8.5 were added. For the trypsin digestion, 0.22 μL of 0.5 μg/μL Trypsin (Waters Corporation, Milford, MA, USA) and 0.56 μL of 0.1 μg/μL LysC which was used to assist trypsin digestion were then added. In some cases, 2.08 μL of 40 ng/μL AspN (Sigma-Aldrich, St. Louis, MO, USA) were used as the digestion enzyme, and the volume of Milli-Q water was adjusted to reach a total volume of 50 μL. The samples were vortexed, centrifuged, and incubated at 37 °C overnight for digestion. Then, 5 μL of 1% trifluoroacetic acid (TFA) were added to quench the reaction. The samples were centrifuged at 18,000 × *g* for 10 min, and the supernatant was used for C18 SpinTip (Thermo Fisher Scientific, Waltham, MA, USA) purification. C18 SpinTip was activated by 20 μL of 0.1% TFA in 80% acetonitrile (ACN) by centrifugation at 1,000 × *g* for 1 min. C18 SpinTip was equilibrated with 20 μL of 0.1% TFA by centrifugation at 1,000 × *g* for 1 min. The tube was replaced, and the sample was loaded to the C18 SpinTip and centrifuged at 1,000 × *g* for 3 min at 4 °C. To remove salts and impurities, the samples were washed twice with 20 μL of 0.1% TFA and, centrifuged at 1,000 × *g* for 1 min at 4 °C each time. The tube was then replaced, and the first peptide elution was performed with 20 μL of 0.1% TFA in 80% ACN by centrifugation at 1,000 × *g* for 1 min at 4 °C. The tube was replaced again for the second peptide elution, using identical conditions. Finally, 5 μL of the eluted samples were dispensed.

### MALDI–MS Analysis

To prepare the peptide calibration standard, P_14_R, ACTH fragment 18–39 (human), and insulin oxidized B chain (bovine) from the MasCal2 kit (Sigma-Aldrich) were diluted with 0.1% or 0.05% trifluoroacetic acid (TFA) as recommended. In addition, insulin (human) MALDI–MS calibrant (Fujifilm Wako Pure Chemical Corporation, Osaka, Japan) was diluted with 10 μL of 0.05% TFA. These solutions were then mixed thoroughly and used as the calibration standard. Then, 2 mg of α-cyano-4-hydroxycinnamic acid (CHCA) were weighed into a tube. Subsequently, 200 μL of 0.1% TFA were added. The solution was then centrifuged, and the supernatant was collected. The matrix solution was mixed with a standard sample or digested rAAV sample in a 1:1 ratio. A 1.5-μL aliquot of each mixture was then deposited onto the FlexMass–DS of MALDI plate (Shimadzu Corporation, Kyoto, Japan) which was inserted into MALDI-8020 instrument (Shimadzu Corporation) to acquire the *m*/*z* spectrum.

### Serotype Identification Algorithm

Serotypes (including their mutant forms) were identified and distinguished by analyzing the *m*/*z* spectra obtained after digestion with specific enzymes. Two analysis modes, designated as “Confirmation mode” and “Identification mode,” were implemented in an in-house Python-based software. The workflows of the software is shown in Figure 1. Confirmation mode evaluates total number of amino acid residues which are matched with amino acid residues solely from the selected serotype. Because this decision metric is based on peptide counts, the procedure is prone to cross-reactivity. Importantly, this mode can confirm whether the enzymatic treatments and MS conditions work. In contrast, Identification mode applies a weighted scoring algorithm that consults a spectral library containing theoretical peptide spectra for serotypes 1–13 and assigns greater weights to serotype-specific peptides before computing a score to nominate the most probable serotype. The identification score *S* is defined as

1 $$S=N\times \overset{N}{\underset{i=1}{\sum }}s_{i}$$

where *N* is the number of identified peptides and *s*_*i*_ denotes the specificity of peptide *i*. Specificity is defined as the smallest absolute *m*/*z* separation between a peptide unique to the target serotype and the nearest theoretical peptide from any nontarget serotype in the library. By construction, this metric down-weights peptides that are vulnerable to small PTM-induced mass shifts or to resolution constraints, and up-weights peptides that remain distinguishable under such perturbations. To reduce matrix-related ambiguity, only peptides with *m*/*z* > 1500 were considered for scoring. The “Variant Identification mode” extends the Identification mode analysis to detect mutant strains. When sequence variants are suspected, activation of the Variant Identification mode triggers an auxiliary routine that evaluates whether the inferred serotype represents a mutant strain. In this mode, when the serotype is identified in the Identification mode, (e.g., 9), the specific peptides are compared individually with the PMFs of other variants (e.g., PHP.eB and GTX) to determine whether it is the original serotype or a variant. Because the algorithm relies on multiple serotype-specific peptides rather than a single epitope, the resulting assay exhibits significantly less cross-reactivity than ELISA, while maintaining high specificity for the target serotype. A detailed step-by-step outline of the workflow and the underlying algorithm executed in Identification mode is provided here:

1. Construction of serotype-amino acid library: The library containing the rAAV serotypes of and their corresponding amino acid sequences was constructed.
2. Calculation of theoretical *m*/*z* for digested peptide: For each serotype to be analyzed, in silico digestion was performed using a specified protease to generate theoretical peptide fragments. The *m*/*z* values of these fragments were calculated. For each peptide, its *m*/*z* value was compared with those of all peptides generated from the in-silico digestion of all serotypes in the library. The minimum *m*/*z* difference between a given peptide and any peptide from other serotypes was calculated as its specificity.
3. Selection of specific peptides: Peptides were ranked in descending order of specificity, and those with specificity distinguishable by the mass spectrometer used were selected as serotype-specific peptides. The total specificity of the selected peptides at this stage exceeded 60. If the total specificity did not reach 60, a different protease was selected, and the analysis was repeated.
4. Peak picking from experimental data: Experimental mass spectrometry data were imported, and peak picking was performed to detect observed peptide peaks.
5. Assignment of detected peaks to serotype peptides: Detected peaks were assigned to theoretical peptide fragments in the library based on their *m*/*z* values.
6. Scoring and serotype identification: For each serotype, the number of assigned fragment peptides (identification number) and the specificity of each assigned peptide were multiplied and summed to generate a score. The serotype in the sample was identified based on this score.

**Figure 1:**
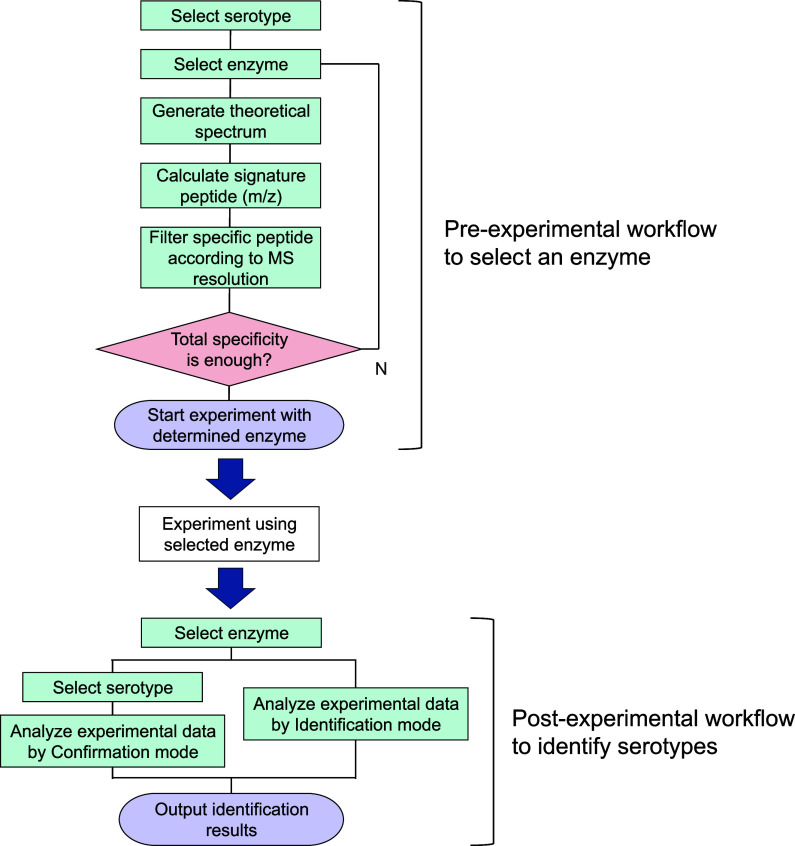
Overview of the in-house software and its application in the experimental workflow. The developed software comprises a pre-experimental enzyme selection algorithm and a postexperimental serotype identification algorithm.

In the step 3, a threshold of 60 was empirically determined, as values above this level consistently resulted in the target serotype being identified as the top hit with good reproducibility. Steps 2–3 and 4–6 are executed automatically upon activating the “Calculate Signature *m/z*” and “Identification Mode” buttons in the software. This automation reduces user error and ensures consistent results across analyses. The respective procedures follow the workflows outlined in Figure 1. These workflows enable reliable and efficient serotype identification, thus highlighting the suitability of the method for research, development, clinical trials, and even marketed stages.

## Results and Discussion

### Enzyme Selection Using in-House Software

Using our in-house software, we performed in silico digests of AAV2, AAV5, AAV6, AAV8, and AAV9 with trypsin and AspN and computed peptide specificity scores; peptides which has specificity >5 are listed in Table 1. Following in silico tryptic digestion, only one serotype-specific peptide was found for AAV6 with modest specificity (5.96), and AAV8 showed the second-lowest cumulative specificity (32.24), indicating that distinguishing AAV6 and AAV8 solely by tryptic PMF would be challenging. In contrast, in silico AspN digestion increased the cumulative specificity for both AAV6 and AAV8 to >60, demonstrating markedly improved discriminative power. Guided by these predictions, we digested AAV2, AAV5, and AAV9 with trypsin and AAV6 and AAV8 with AspN, and then acquired PMFs by MALDI–MS.

**Table 1:** Signature Peptides and Their Specificity for Each Serotype^a^

Enzyme	Serotype	Signature peptide (Averaged mass)	Specificity
Trypsin	2	LNFGQTGDADSVPDPQPLGQPPAAPSGLGTNTMATGSGAPMADNNEGADGVGNSSGNWHCDSTWMGDR (6760.2)	25.98
		TTNPVATEQYGSVSTNLQR (2067.2)	14.86
		MAADGYLPDWLEDTLSEGIR (2253.5)	13.00
		QAATADVNTQGVLPGMVWQDR (2258.5)	7.97
		RPVEHSPVEPDSSSGTGK (1867.0)	5.18
	5	MELEGASYQVPPQPNGMTNNLQGSNTYALENTMIFNSQPANPGTTATYLEGNMLITSESETQPVNR (7165.9)	101.21
		TEEDSKPSTSSDAEAGPSGSQQLQIPAQPASSLGADTMSAGGGGPLGDNNQGADGVGNASGDWHCDSTWMGDR (7179.4)	87.63
		EFLGLEAGPPKPKPNQQHQDQAR (2586.9)	49.12
		SGSVDGSNANAYFGYSTPWGYFDFNR (2881.0)	42.17
		NTPVPGNITSFSDVPVSSFITQYSTGQVTVEMEWELK (4090.5)	39.04
		WNPEIQYTNNYNDPQFVDFAPDSTGEYR (3382.5)	27.07
		EHDISYNEQLEAGDNPYLK (2236.4)	17.13
		VAYNVGGQMATNNQSSTTAPATGTYNLQEIVPGSVWMER (4145.6)	15.98
		IPETGAHFHPSPAMGGFGLK (2052.4)	14.86
		TGNNFEFTYNFEEVPFHSSFAPSQNLFK (3300.6)	9.13
		EVTVQDSTTTIANNLTSTVQVFTDDDYQLPYVVGNGTEGCLPAFPPQVFTLPQYGYATLNR (6673.4)	8.69
	6	FGTVAVNLQSSSTDPATGDVHVMGALPGMVWQDR (3546.0)	5.96
	8	SSFYCLEYFPSQMR (1759.0)	13.21
		TIANNLTSTIQVFTDSEYQLPYVLGSAHQGCPPPFPADVFMIPQYGYLTLNNGSQAVGR (6405.2)	10.04
		LNFGQTGDSESVPDPQPIGEPPAGPSGLGSGTMAAGGGAPMADNNEGADGVGSSSGNWHCDSTWLGDR (6600.9)	8.99
	9	TTNPVATESYGQVATNHQSAQAQAQTGWVQNQGILPGMVWQDR (4671.1)	141.18
		QISNSTSGGSSNDNAYFGYSTPWGYFDFNR (3341.4)	13.99
		TIANNLTSTVQVFTDSDYQLPYVLGSAHEGCLPPFPADVFMIPQYGYLTLNDGSQAVGR (6395.2)	10.04
		LNSFITQYSTGQVSVEIEWELQK (2701.0)	9.01
		LNFGQTGDTESVPDPQPIGEPPAAPSGVGSLTMASGGGAPVADNNEGADGVGSSSGNWHCDSQWLGDR (6682.0)	8.69
AspN	6	DEEEIKATNPVATERFGTVAVNLQSSST (2995.2)	34.02
		DGHFHPSPLMGGFGLKHPPPQILIKNTPVPANPPAEFSATKFASFITQYSTGQVSVEIEWELQKENSKRWNPEVQYTSNYAKSANV (9585.8)	23.04
		DTSFGGNLGRAVFQAKKRVLEPFGLVEEGAKTAPGKKRPVEQSPQEP (5053.7)	5.96
	8	DWQRLINNNWGFRPKRLNFKLFNIQVKEVTQNEGTKTIANNLTSTIQVFT (5938.8)	27.03
		DVFMIPQYGYLTLNNGSQAVGRSSFYCLEYFPSQMRRTGNNFEFSYQFE (5805.4)	24.99
		DTSFGGNLGRAVFQAKKRVLEPLGLVEEGAKTAPGKKRPVEPSPQRSP (5102.9)	14.03
		DQYLYYLSRTQSTGGTAGTQQLLFSQAGPNNMSAQAKNWLPGPCYRQQRVSTTLSQNNNSNFAWTGATKYHLNGR (8362.2)	14.03
		DEERFFPSSGVLMFGKQGAGK (2288.6)	7.14

a (*m/z* > 1500 and specificity >5.0)

### MALDI–MS Analysis of AAV Digested Peptides

The tryptic *m*/*z* spectra for AAV2, AAV5, and AAV9 are shown in Figure 2A–C, and the AspN *m*/*z* spectra for AAV6 and AAV8 are presented in Figure 2D,E. Figure 2F,G shows the *m*/*z* spectra obtained following trypsin digestion of AAV9 variants, AAV-PHP.eB and AAV.GTX. In Figure 2H, all *m*/*z* spectra are overlaid, revealing that *m*/*z* signals below 1500 include shared signals originating from the matrix. These observations reveal a limitation of MALDI–MS-based spectral acquisition, i.e., it is challenging to include digested peptides with *m*/*z* < 1500 in the scoring process. In our method, high-specificity peptides tended to occur at higher *m*/*z*, so incorporating peptide specificity into the scoring scheme mitigated the impact of excluding low-*m*/*z* peptides on identification outcomes.

**Figure 2:**
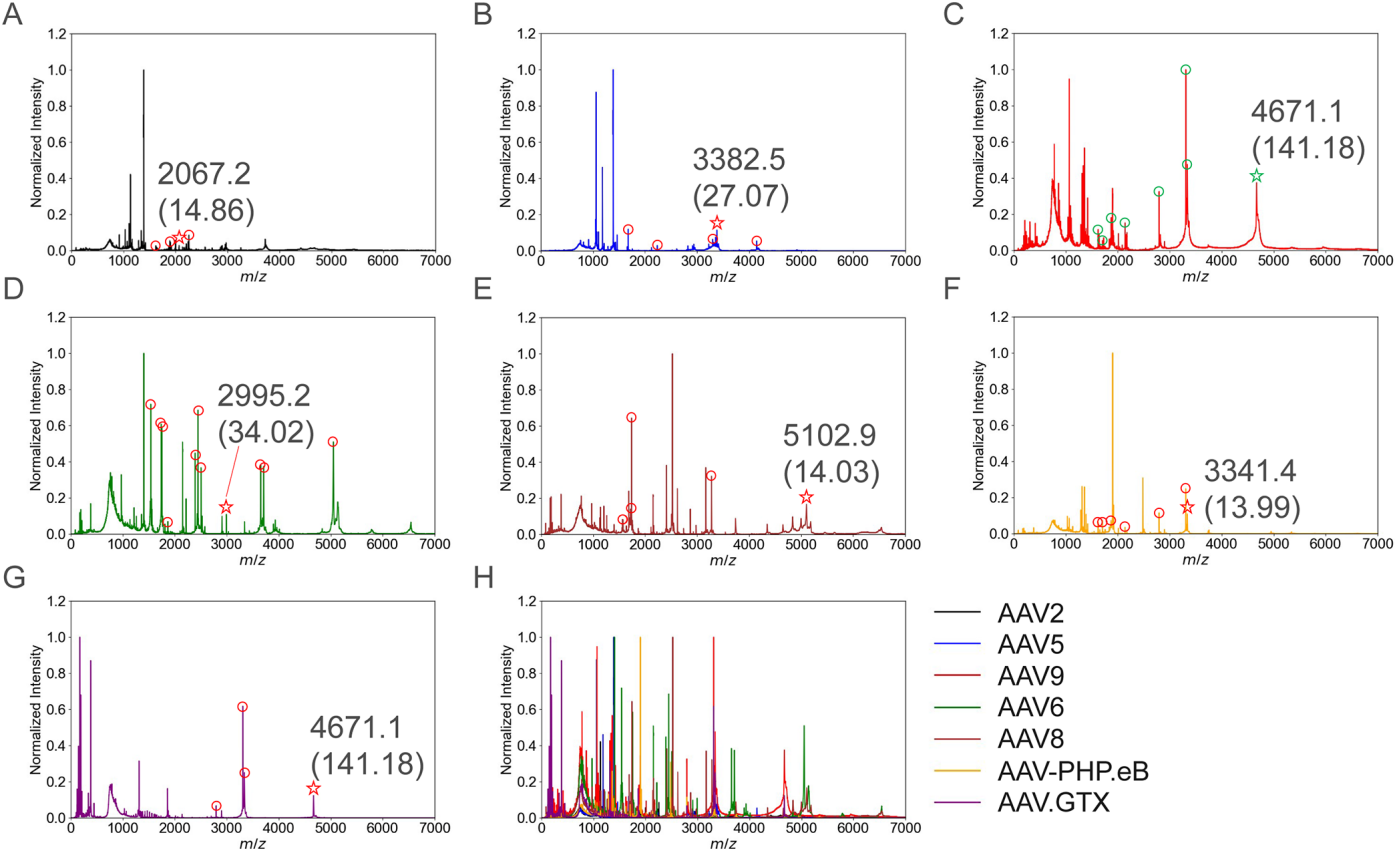
MALDI–MS spectra of rAAVs treated with the selected enzymes. (A, B, C) *m*/*z* spectra of AAV2, AAV5, and AAV9 digested with trypsin, respectively. (D, E) *m*/*z* spectra of AAV6 and AAV8 digested with AspN, respectively. (F, G) *m*/*z* spectra of AAV9 variants, AAV-PHP.eB and AAV.GTX, digested with trypsin. (H) overlay of all spectra, highlighting serotype-specific peptides with distinct *m*/*z* values that were experimentally validated. Peaks corresponding to identified peptides are indicated with circular markers. For each identified serotype, the peptide exhibiting the highest specificity is highlighted with a star symbol, and its experimental *m*/*z* value (specificity) is explicitly annotated. A complete list of all identified peptides and their corresponding *m*/*z* values is shown in Table S1.

The analysis of Figure 2A–G using the in-house software is summarized in Figure 3. In Confirmation mode, peptides comprising a total of approximately 100 amino acid residues were detected for all serotypes, suggesting protease cleavage worked well for each serotype. Subsequently, in Identification mode, all serotypes, including variants, were successfully identified (refer to the “Identification test” and “Variant test” columns in Figure 3). Consistent with their close similarity to AAV9, AAV-PHP.eB and AAV.GTX were first called as AAV9 by the Identification mode. After activating the Variant Identification mode, the algorithm correctly resolved them as AAV-PHP.eB and AAV.GTX, respectively. These results highlight the practical utility of MALDI–MS in serotype identification, showcasing its simultaneous simplicity and reliability. For example, 5 peptides were detected for AAV5 with *m*/*z* values of 1678.8, 2236.4, 3300.6, 3382.5, and 4145.6 and specificity scores of 1.07, 17.13, 9.13, 27.07, and 15.98, respectively. Thus, the identification score for AAV5 was calculated as 351.9 by multiplying the number of peptides identified above a predefined threshold by the sum of their specificity scores, as defined in (eq 1). Similarly, for AAV9, a peptide was detected with *m*/*z* = 4671.1 and a high specificity score of 141.18, enabling identification with a remarkably high score. In all cases, the top-hit serotype matched the serotype used experimentally, although other serotypes occasionally showed moderate scores. Misidentification risk was low overall under our test conditions and was oxidation-contingent for AAV.GTX (see “Robustness of MALDI–MS-based identification methods”). Post-translational modifications (PTMs) introduced during sample preparation—such as methionine oxidation or asparagine deamidation—occasionally diversified peak patterns and increased off-target scores. Notably, MALDI ionization tends to generate predominantly singly charged ions, which reduces spectral complexity from PTM-related peak families compared with methods dominated by multiply charged species. In addition, and by definition of the specificity metric, peptides less susceptible to PTM-induced mass shifts or resolution limitations are assigned higher specificity scores. Together, these findings support the effectiveness of MALDI–MS in providing straightforward spectra and reducing misidentifications associated with preparation-induced artifacts.

**Figure 3:**
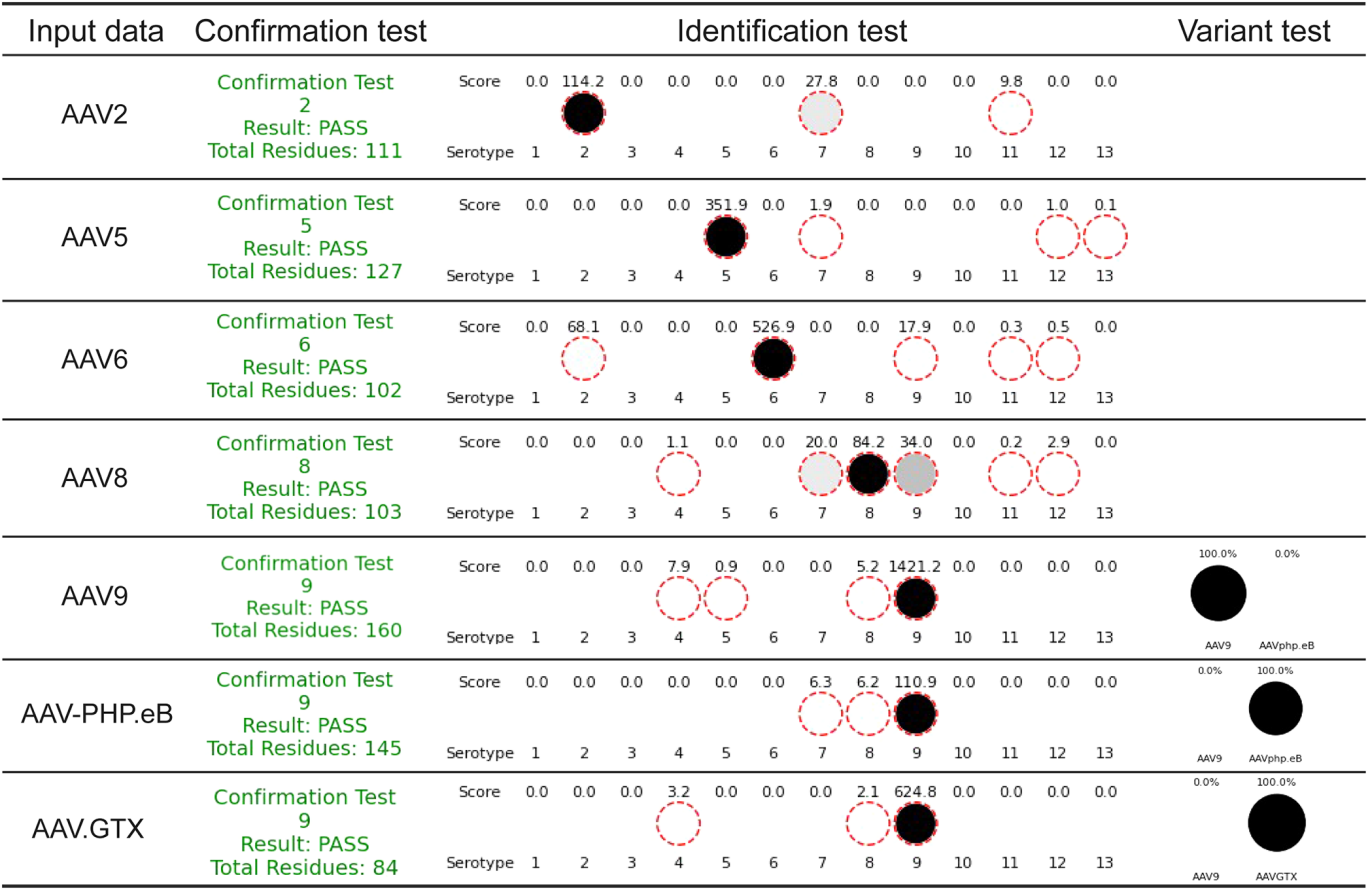
Analysis of experimental data using in-house software. Summary of results obtained in Confirmation and Identification modes: AAV2, AAV5, AAV9, AAV-PHP.eB, and AAV.GTX were digested by trypsin, whereas AAV6 and AAV8 were digested by AspN. All serotypes passed the confirmation test, and the correct serotype was returned as the top hit in the identification test. The Variant Identification mode distinguished the variants, AAV-PHP.eB or AAV.GTX, from AAV9.

### Identification of AAV9 Variants Using in-House Software

It is known that even 10- or 2-residues changes can markedly alter biological properties. For example, AAV-PHP.eB differs from the parent AAV9 at ten residues: a K449R substitution, a double substitution at positions 587/588 (A587D and Q588G), and insertion of the heptapeptide TLAVPFK immediately after residue 588 within the VR-VIII loop shown in Figure 4A. The peptide insertion is within the VR-VIII loop, which is implicated in the vector’s enhanced tropism for the central nervous system.^33–35^ AAV.GTX has Y446F and Y731F mutations (Figure 4A) to prevent phosphorylation and ubiquitination within cells, thereby avoiding degradation,^36^ resulting in widespread gene delivery to the brain and spinal cord in vivo in mice.^37^

**Figure 4:**
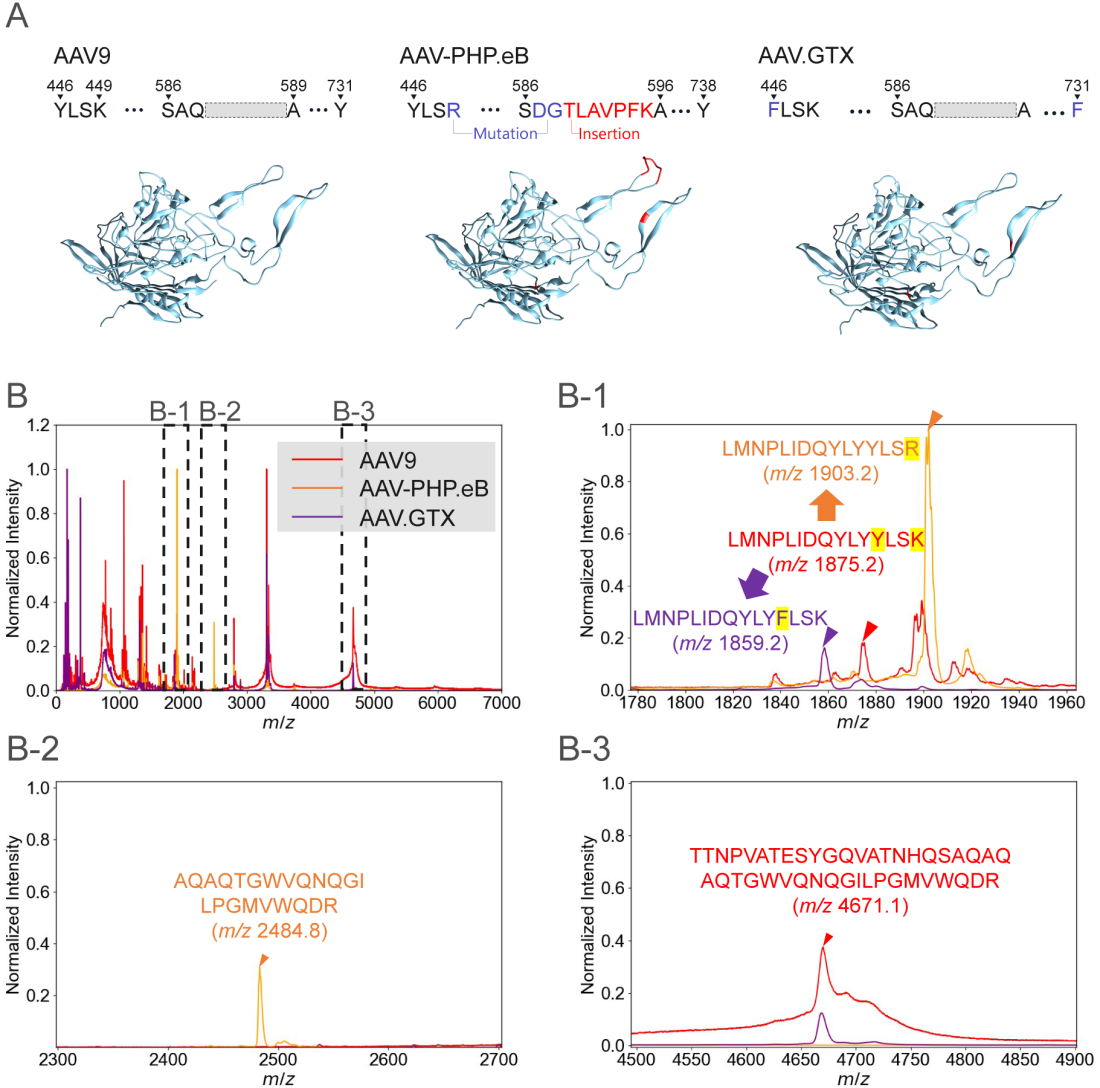
Identification of the AAV9 variants AAV-PHP.eB and AAV.GTX. (A) Mutated residues of AAV-PHP.eB and AAV.GTX relative to AAV9 and AlphaFold2-predicted capsid structures of AAV9, AAV-PHP.eB, and AAV.GTX, where regions that differ from the AAV9 sequence are highlighted in red. AAV-PHP.eB has 10 amino acids different from AAV9, including three-point mutations (blue) and a seven-residue insertion (red). AAV.GTX contains two-point mutations (blue) relative to AAV9. (B) Tryptic peptides derived from the capsids of AAV9, AAV-PHP.eB, and AAV.GTX were analyzed by MALDI–MS. The enlarged view in (B-1) shows the detection of variant-specific peptides for all three vectors, whereas (B-2) and (B-3) highlight peptide peaks unique to AAV-PHP.eB and AAV9, respectively.

Antibody-based assays may fail to resolve such subtle sequence differences and can exhibit antibody-dependent cross-reactivity or false negatives: in a dot blot using the ADK9 antibody from an AAV9 ELISA kit (Progen, Heidelberg, Germany) as shown in Figure S1, AAV-PHP.eB was not detected, whereas AAV.GTX produced a positive signal, indicating cross-reactivity with ADK9. Moreover, to our knowledge, there is currently no off-the-shelf consumable antibody that specifically recognizes AAV-PHP.eB; consequently, MALDI–MS is practically required for identity testing of this variant. We then applied our methods to AAV-PHP.eB and AAV.GTX.

Although the overall *m*/*z* patterns of AAV9, AAV-PHP.eB, and AAV.GTX are highly similar owing to their shared sequences, analysis of variant-specific peptides using MALDI–MS enable unambiguous identification as shown in Figure 3. To better understand these differences, the theoretical tryptic peptides resulting from these mutations were analyzed. As shown in Figure 4A, the mutations in AAV-PHP.eB lead to the replacement of the theoretical tryptic peptide “LMNPLIDQYLYYLSK” (*m*/*z* 1875.2) of AAV9 with “LMNPLIDQYLYYLSR” (*m*/*z* 1903.2). Furthermore, the double mutation and heptapeptide insertion are expected to split the parent peptide “TTNPVATESYGQVATNHQSAQAQAQTGWVQNQGILPGMVWQDR” (*m/*z 4671.1) into two fragments: “TTNPVATESYGQVATNHQSDGTLAVPFK” (*m*/*z* 2935.2) and “AQAQTGWVQNQGILPGMVWQDR” (*m*/*z* 2484.8). Similarly, in AAV.GTX, the parent peptide “LMNPLIDQYLYYLSK” (*m*/*z* 1875.2) is altered to “LMNPLIDQYLYFLSK” (*m*/*z* 1859.2) because of the Y446F mutation. The tryptic peptide derived from the Y738F mutation has *m*/*z* < 1500 and was therefore excluded from this analysis.

Overlaid *m*/*z* spectra of tryptic peptides from AAV9, AAV-PHP.eB, and AAV.GTX are shown in Figure 4B. Because of the high sequence similarity of these serotypes (more than 98%), the *m*/*z* spectra in Figure 4B show a higher degree of overlap. Figure 4B-1,B-2,B-3 show expanded views of Figure 4B. Figure 4B-1 confirms the presence of the variant-specific peaks at *m*/*z* 1903.2 (AAV-PHP.eB) and 1859.2 (AAV.GTX), which are absent from the AAV9 spectrum. Figure 4B-2 reveals the detection of the *m*/*z* 2484.8 peak specific to AAV-PHP.eB, and Figure 4B-3 confirms the detection of the *m*/*z* 4671.1 peak, a unique peptide of AAV9 and AAV.GTX without heptapeptide insertion. These results demonstrate that even closely related rAAV serotypes, such as AAV9, AAV-PHP.eB, and AAV.GTX, can be distinguished by analyzing their specific tryptic peptides using MALDI–MS in a straightforward and efficient manner.

### Robustness of MALDI–MS-Based Identification

For the practical application, it is necessary whether sample concentration effects the identification results. Figure S2 summarizes a concentration-dependence study of AAV9. Within the range of 7.0 × 10^10^ to 5.6 × 10^11^ capsids, all samples were consistently and correctly identified, demonstrating a wide concentration range in which the method remains robust. At higher concentrations, the *m*/*z* spectra became increasingly congested, indicating higher misassignment risks. Thus, a working concentration of 7.0 × 10^10^ to 2.8 × 10^11^ capsids is recommended for routine analyses.

Additionally, the peptides preparation steps in the protocol can inadvertently introduce PTMs in the rAAV capsid proteins. Therefore, it is crucial to ensure that these potential artifacts do not compromise the reliability of the MALDI–MS-based identification assay. We then assessed reproducibility across different operators, on different days, and using different SpinTips (Figure S3), and we present representative identifications in the main text (Figure 3). When summarizing results from both figures (Figure 3 *n* = 1; Figure S3, *n* = 3 per serotype), the identification success rates were as follows: AAV9 in Identification mode, 100% (4/4 runs); AAV-PHP.eB in Variant Identification mode, 100% (4/4 runs); and AAV.GTX in Variant Identification mode, 50% (2/4 runs). Notably, although AAV.GTX was correctly called in the single run shown in Figure 3, only one of the three independent repeats in Figure S3 succeeded. Misassignments for AAV.GTX occurred specifically in runs where methionine oxidation of the GTX-specific peptide LMNPLIDQYLYFLSK (*m*/*z* 1859.2) produced an oxidized species at *m*/*z* 1875.2 that overlaps the AAV9-specific peptide LMNPLIDQYLYYLSK (*m*/*z* 1875.2), leading to incorrect calls (Figure S4A). Thus, AAV.GTX errors were oxidation-contingent rather than intrinsic to the workflow; in runs where oxidation was absent or minimal, AAV.GTX was correctly resolved. Although the interference caused by oxidized peptide forms is a phenomenon particularly prominent in the Variant Identification mode, where the number of specific peptides is inherently reduced, this issue can be mitigated through appropriate algorithmic adjustments. For specific peptides containing methionine, we implemented a logic that checks whether both the unoxidized peptide and its oxidized form (+16 Da) are detected (Figure S4B). When simultaneous detection occurs, any peptide whose *m*/*z* corresponds to the oxidized form is redefined as nonspecific. This correction allows accurate identification of the GTX serotype even when oxidation has occurred. However, while such algorithmic modifications can improve robustness, increasing the number of PTMs considered inevitably reduces the pool of specific peptides and complicates the identification workflow. This may ultimately increase the risk of misidentification. Therefore, from the standpoint of reliable serotype discrimination, minimizing PTMs such as oxidation during sample preparation remains the most desirable and fundamental approach.

Furthermore, we confirmed that the method also correctly identified commercially sourced rAAV samples purchased from VectorBuilder (Chicago, IL), demonstrating that the workflow is applicable not only to in-house preparations but also to externally manufactured vectors as shown in Figure S5.

## Conclusions

This study experimentally evaluated the feasibility of serotype identification using MALDI–MS. The serotypes AAV2, AAV5, and AAV9 were identified using trypsin digestion, with scores exceeding those of the second-highest serotype by more than 4-fold. In-house software was used for in silico analysis, which confirmed that AAV6 and AAV8 did not yield sufficiently specific peptides with trypsin digestion. Therefore, AspN digestion was employed for these serotypes, which enabled successful identification. The AAV9 variants, AAV-PHP.eB and AAV.GTX, which share over 98% amino acid sequence identity with AAV9 were also investigated. These variants were distinguished by examining the differences in one to three peptides resulting from mutations in their PMFs. The findings confirmed that MALDI–MS is a powerful tool for serotype identification, with advantages over established methods. Compared with ELISA, MALDI–MS offers lower cross-reactivity and can differentiate even few-residue variants. Additionally, it eliminates the need for antibodies, which are often derived from animals and pose challenges in terms of stability and management. Another significant benefit is the reduced cost; Although MALDI instruments are more than one-third less expensive than high-resolution Orbitrap systems, they remain more costly than ELISA plate readers. However, because per-analysis consumable costs for MALDI are approximately an order of magnitude lower, the total cost of ownership tends to favor MALDI when the analytical scale exceeds approximately one hundred measurements as shown in Table S2. Although MALDI–MS lacks the quantitative capabilities of ELISA, viral genome quantification via PCR, combined with full/empty ratio evaluation using mass photometry, charge detection mass spectrometry, or analytical ultracentrifugation^38^ can indirectly estimate capsid quantities during clinical phases. Compared with LC–MS/MS, MALDI–MS eliminates the need for liquid chromatography and the operation of high-resolution MS workflows, dispensing with complex gradient optimization, capillary-column management, and retention-time library maintenance, while offering approximately a 10-fold reduction in sample consumption. However, MALDI–MS cannot achieve high sequence coverage or support PTM analysis to the same extent as LC–MS/MS. Therefore, during drug discovery stages where complete sequence characterization and PTM evaluation are critical, LC–MS/MS remains the preferred method.

In conclusion, serotype identification using MALDI–MS is a simple, cost-effective, and reliable method with minimal cross-reactivity. Although MALDI–MS requires a certain level of expertise in mass spectrometry operation and data interpretation, it still serves as an orthogonal approach to established methods such as ELISA and LC–MS/MS and is well-suited for research, development, clinical testing, and commercial phase. Moreover, the methodological framework established herein may also inform serotype or strain typing of viral vectors besides rAAV, including lentiviruses and oncolytic adenoviruses. This broad applicability contributes to the diverse field of viral vector-based gene therapy.

## Supplementary Material



## Notes

Additional figures, including experimental data that support the claims of this paper (PDF)
