# Supplementary material for: Identification of Recombinant Adeno-Associated Virus Serotypes by Matrix-Assisted Laser Desorption/Ionization Mass Spectrometry
Source: Anal Chem. 2026 Mar 1;98(10):7170–9. doi: 10.1021/acs.analchem.5c05430 (PMC13000878; doi:10.1021/acs.analchem.5c05430)
Supplement: Supplementary file 1 [file ac5c05430_si_001.pdf]

Supporting Information

Identification of Recombinant Adeno-Associated Virus Serotypes by Matrix-Assisted Laser Desorption/Ionization Mass Spectrometry

Ryoji Nakatsuka[1, 2, 3], Kenjiro Matsumoto[4], Yannan Liu[1], Kimitoshi Takeda[4], Yasuo Tsunaka[1], Tetsuo Torisu[1], Yuki Yamaguchi[1], and Susumu Uchiyama[1*]

[1]Department of Biotechnology, Graduate School of Engineering, The University of Osaka, 2-1 Yamadaoka, Suita, Osaka 565-0871, Japan

[2]Technology Research Laboratory, Shimadzu Corporation, 1 Nishinokyo-Kuwabaracho, Nakagyo-ku, Kyoto 604-8511, Japan

[3]Osaka University Shimadzu Analytical Innovation Research Laboratories, The University of Osaka, 2-1 Yamadaoka, Suita, Osaka 565-0871, Japan

[4]U-Medico Inc., 2-1 Yamadaoka, Suita, Osaka 565-0871, Japan

* Corresponding author: Susumu Uchiyama, Ph.D. E-mail: suchi@bio.eng.osaka-u.ac.jp

Contents

Abstract for Supporting Information. 3

m/z value of identified peptides for each serotype in Figure 2 3

Dot blot analysis of AAV9, AAV-PHP.eB, and AAV.GTX 4

Concentration-dependence study of AAV9 5

Reproducibility of Identification mode and Variant identification mode using AAV9, AAV-PHP.eB, and AAV.GTX 6

Impact of Peptide Oxidation on the Reproducibility of AAV.GTX Identification. 7

Identification test of commercially available rAAV by MALDI-MS 8

Comparison table of ELISA and MALDI-MS 9

Abstract for Supporting Information.

We have added Figures mentioned in the main text as follow. The order of the contents in this information correspond to the order described in the main text.

***m/z* value of identified peptides for each serotype in Figure 2**

Table S1: *m/z* value, total specificity, and number of identified peptides for each serotype in Figure 2

Serotype	2	5	6	8	9	PHP.eB	GTX
m/z value	1903.2<br>2258.5<br>2067.2<br>1621.8<br>1872.2	3382.5<br>3300.6<br>2236.4<br>1678.8<br>4145.6	1868.1<br>3656.0<br>2506.9<br>1540.8<br>5053.7<br>1739.0<br>2995.2<br>2392.5<br>2447.8<br>3712.0<br>1563.7<br>1754.9	1718.8<br>3278.8<br>1739.0<br>5102.9<br>1575.7<br>1563.7	1621.8<br>2796.0<br>1875.2<br>2141.5<br>4671.1<br>3341.4<br>3310.5<br>1706.9<br>1872.2	1621.8<br>2796.0<br>2141.5<br>3341.4<br>3310.5<br>1706.9<br>1872.2	3341.4<br>3310.5<br>2796.0<br>4671.1
Total specificity	22.83	70.38	43.91	14.03	157.91	15.84	156.20
Peptide number	5	5	12	6	9	7	4

Dot blot analysis of AAV9, AAV-PHP.eB, and AAV.GTX

Dot blot analysis was performed to detect AAV9 and its variants, AAV-PHP.eB and AAV.GTX, using a biotinylated ADK9 antibody from the AAV9 ELISA kit (Progen, Heidelberg, Germany). AAV9 from the same batch as used in the main experiments served as the positive control, while AAV9 incubated at 95°C for 10 minutes was used as the negative control. AAV-PHP.eB was not detected due to mutations in the variable region of VP3, the epitope recognized by ADK9. In contrast, AAV.GTX was detected similarly to AAV9. These findings suggest that ADK9 cross-reacts with the mutant AAV.GTX capsid, even though it has not been reported to cross-react with wild-type variants.

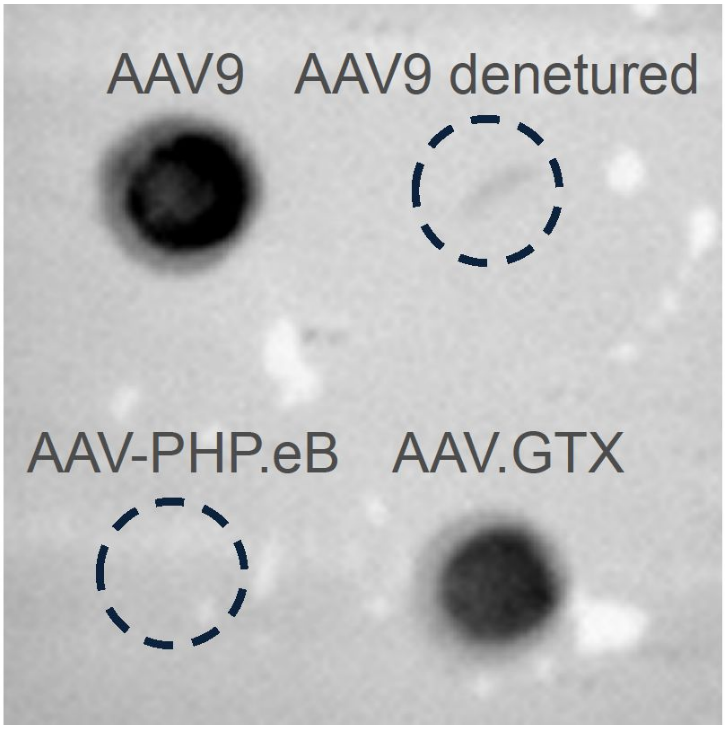

Figure S1. Dot blot analysis of AAV9, AAV-PHP.eB, and AAV.GTX using ADK9 antibody. It was revealed that AAV9 and AAV.GTX were similarly detected, whereas AAV-PHP.eB, which carries mutations in the recognized epitope, were not detected.

Concentration-dependence study of AAV9

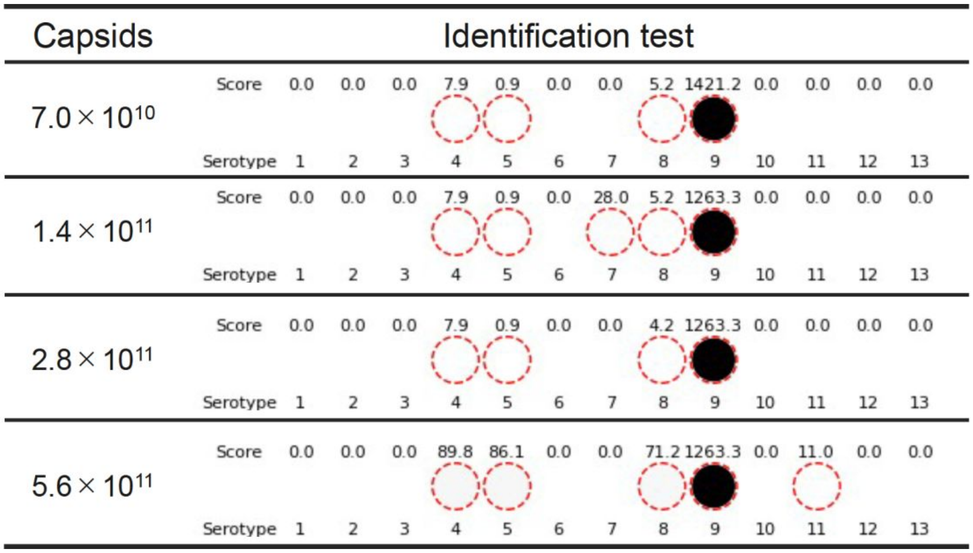

Figure S2. Concentration dependence study of AAV9. AAV9 stock at 2.33×10^{13} cp/mL was diluted 1-, 2-, 4-, and 8-fold. For each dilution, 24 µL contained 5.6×10^{11}, 2.8×10^{11}, 1.4×10^{11}, and 7.0×10^{10} capsids, respectively. Each sample was analyzed by MALDI–MS, and the spectra were processed with in-house software, which consistently identified AAV9.

Reproducibility of Identification mode and Variant identification mode using AAV9, AAV-PHP.eB, and AAV.GTX

Reproducibility of AAV9, AAV-PHP.eB, and AAV.GTX was assessed by multiple experiments performed on different days by two investigators (R.N. and K.M.) using distinct SpinTips. Both AAV9 and AAV-PHP.eB yielded consistent identification results in both Identification mode and Variant Identification mode. In contrast, while AAV.GTX results were reproducible in Identification mode, they were detected in 2 out of 4 runs in Variant Identification mode, indicating lower reproducibility.

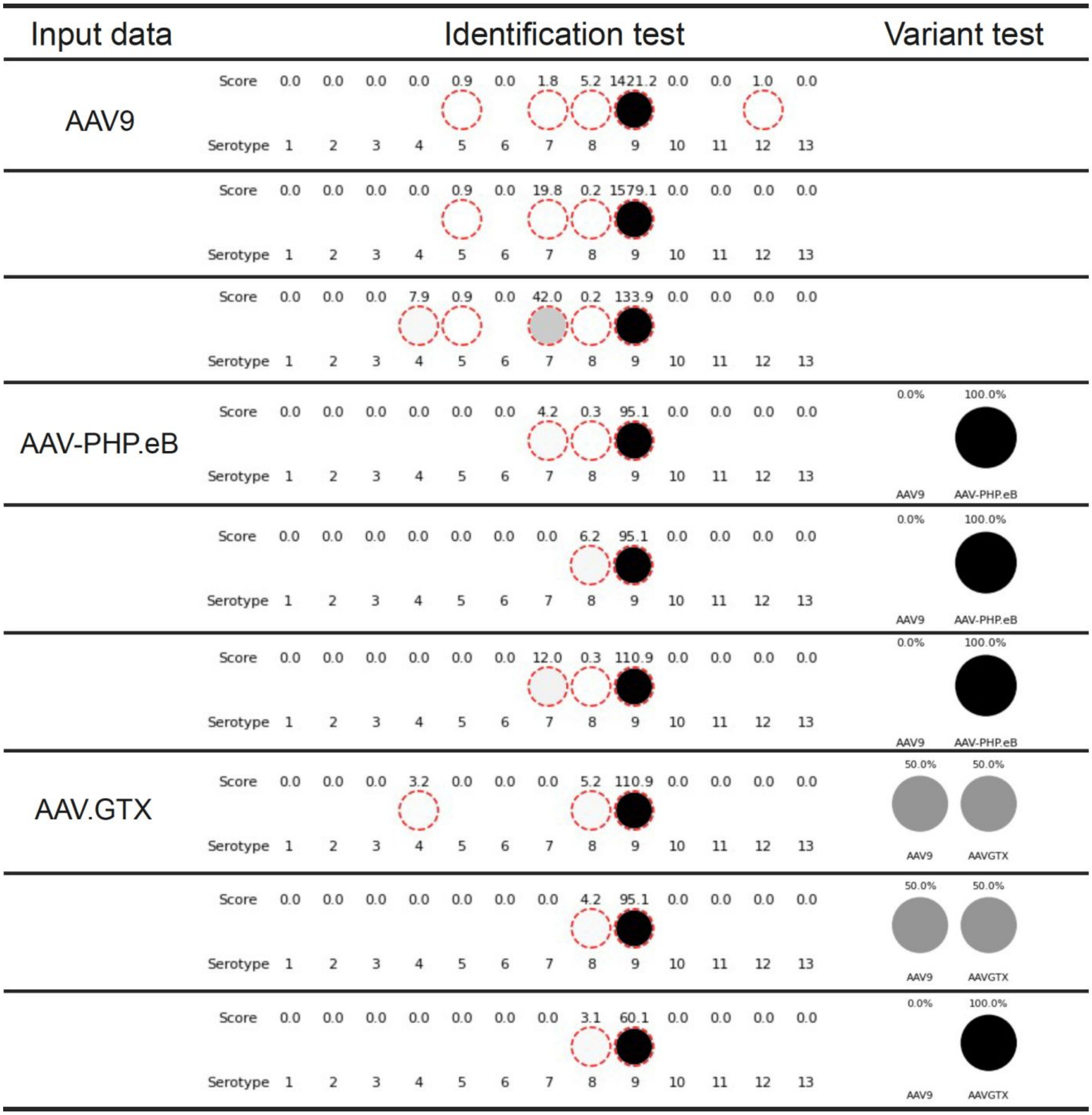

Figure S3. Reproducibility of Identification mode and Variant identification.

Impact of Peptide Oxidation on the Reproducibility of AAV.GTX Identification.

Focusing on the low reproducibility of AAV.GTX in Variant Identification mode, we found that the oxidized form of the AAV.GTX-specific peptide LMNPLIDQYLYFLSK (*m/z* = 1859.2) was misidentified as the AAV9-specific peptide LMNPLIDQYLYYLSK (*m/z* = 1875.2). Consequently, in approximately half of the analyses, the peptide was assigned ambiguously between AAV9 and AAV.GTX. When indicated as "Identified" (purple line), the peak intensity of the oxidized LMNPLIDQYLYFLSK was sufficiently low to be excluded from peak processing, enabling correct identification of AAV.GTX. Such interference of specific peptides caused by PTMs can be resolved by introducing an additional correction step in the scoring logic: when both X and X+16 are assigned, any specific peptide whose m/z corresponds to the oxidized form is excluded from the specific peptide set.

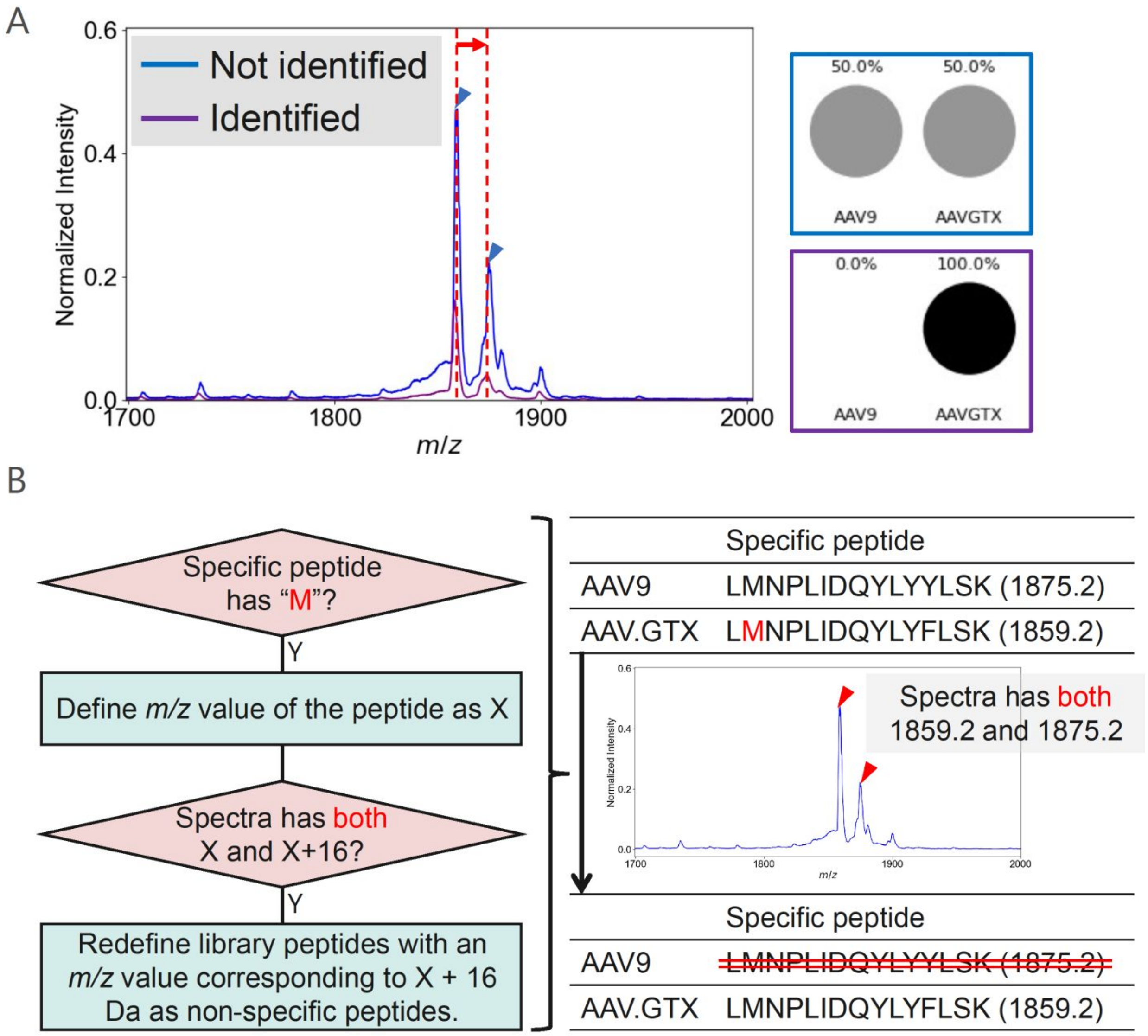

Figure S4. Challenges in identification arising from interference by oxidized forms of AAV.GTX–specific peptides and the corresponding mitigation strategy. (A) *m/z* spectra of AAV.GTX when correctly identified versus not identified and identification results using Variant Identification mode. (B) When both the unoxidized and oxidized peptides are detected, the algorithm enables correct identification even for oxidized AAVs by excluding the *m/z* of the oxidized peptide from the set of specific peptides.

Identification test of commercially available rAAV by MALDI-MS

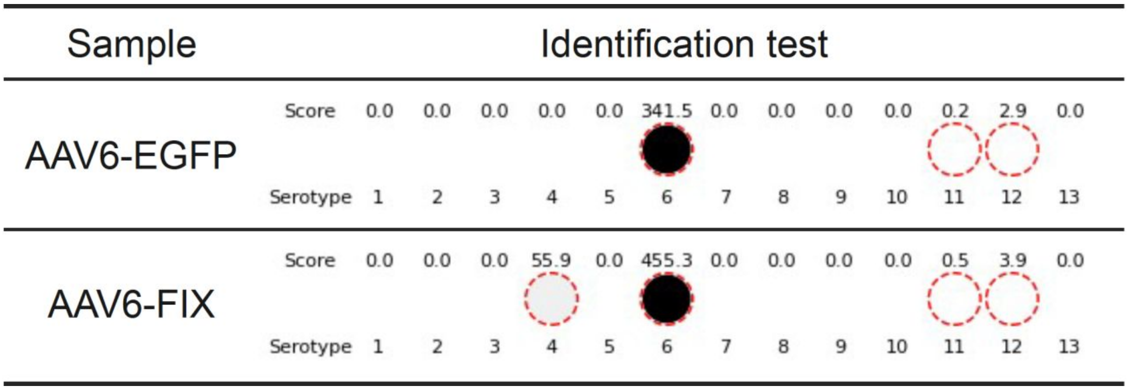

Figure S5. Identification test of commercially available rAAV by MALDI-MS. AAV6-EGFP and AAV6-FIX purchased from VectoBuilder yielded serotype identification results comparable to those obtained from in-house rAAVs, demonstrating that the method performs equivalently on commercially sourced materials.

Comparison table of ELISA and MALDI-MS

Table S2. Comparison table of ELISA and MALDI-MS.

Contents		ELISA	MALDI-MS
Performance	Cross-reactivity	Many reports	None
	Time to analysis	Hands on time: ~2 hours	Incubation: over night<br>Hands on time: ~30 min
	Regent dependence	High	None
Cost	Initial installation	Medium	High
	Operational	High	Low
	For above 115 analyses	Higher	Lower
